# Bridging Effective Stress and Soil Water Retention Equations in Deforming Unsaturated Porous Media: A Thermodynamic Approach

**DOI:** 10.1007/s11242-017-0837-9

**Published:** 2017-03-04

**Authors:** J. M. Huyghe, E. Nikooee, S. M. Hassanizadeh

**Affiliations:** 1 Bernal Institute, University of Limerick, Limerick, Ireland; 2 Department of Mechanical Engineering, Eindhoven University of Technology, Eindhoven, The Netherlands; 3 Earth Sciences Department, Utrecht University, Utrecht, The Netherlands; 4 Civil and Environmental Engineering Department, School of Engineering, Shiraz University, Shiraz, Iran

**Keywords:** Porous medium, Wetting fluid, Non-wetting fluid, Saturation

## Abstract

The finite deformation of an unsaturated porous medium is analysed from first principles of mixture theory. An expression for Bishop’s effective stress is derived from (1) the deformation-dependent Brooks and Corey’s water retention curve and (2) the restrictions on the constitutive relationships of an unsaturated medium subject to finite deformation. The resulting expression for the effective stress parameter $\chi$ is reasonably consistent with experimental data from the literature. Hence, it is shown that Bishop’s equation is constitutively linked to water retention curves in deforming media.

## Introduction

Unsaturated soil mechanics is of utmost importance in many applications. Examples are rainfall-induced landslides and slope failures, the settlement of foundations on (and bearing capacity of) the unsaturated soils, and wetting- and drying-induced volume changes in the expansive and collapsible soils. This explains why the mechanical behaviour of unsaturated soils has been a subject of interest in recent decades.

In order to construct a fundamental framework for modelling the mechanical behaviour of unsaturated soils, a clear understanding of coupling between hydraulics and stress states of unsaturated soils is necessary. On the one hand, the volume fraction of different phases (air, water and solid) appears in the definition of stress measures (stress state variables). On the other hand, the variation of volume fractions themselves depends on the current stress level in a deformable unsaturated porous medium. This coupling is commonly referred to as hydromechanical coupling. Recent experimental studies in unsaturated soil mechanics have pointed to such coupling. Also, a number of modelling studies have tried to take it into account (Gallipoli et al. 2003a, b; Sun et al. 2007, 2008; Sheng and Zhou 2011; Zhou et al. 2012; Sun and Sun 2012; Casini et al. 2013). The hydromechanical coupling frameworks introduced to date for unsaturated soils are mainly of an empirical nature, and the need for a rigorous thermodynamic framework to address the hydromechanical coupling is still being felt. As the term hydromechanical coupling explicitly points out, it is a two-way coupling. Therefore, an essential question is that as one can measure and obtain the volume fraction of different phases in unsaturated soils at certain stress levels, how to formulate the stress state variables which account for the stress-level dependency of the amount of different phases. A comprehensive formulation for this purpose can only be obtained based on the principles of thermodynamics, by means of balance laws, and through a rigorous mathematical framework rather than a heuristic approach. The starting point is to understand how volume fractions of different phases change in an unsaturated soil and how they can be defined based on other physical state variables. Moreover, it is necessary to know how such relationship is affected by the stress level. One major characteristic of unsaturated soils is the soil water retention curve (SWRC), which specifies capillary pressure value associated with the water saturation degree. In recent years, experimental evidences have clearly shown that soil water retention curves are dependent on the stress level (Romero and Vaunat 2000; Karube and Kawai 2001; Gallipoli et al. 2003b; Tarantino and Tombolato 2005; Nuth and Laloui 2008; Tarantino 2009; Uchaipichat 2010). Tarantino (2009) has considered the dependency of air entry value on stress level and has offered a SWRC equation for deformable media. He has modified the van Genuchten equation for SWRC to account for soil deformation and SWRC dependency on the stress level. Van Genuchten equation, in its original form, reads (Genuchten 1980):

1 $$\begin{aligned} \varTheta =\left( \frac{1}{1+(\alpha p_\mathrm{c})^{n}}\right) ^m \end{aligned}$$

where $\alpha$, *n* and m are fitting parameters and $\varTheta =\frac{S_\mathrm{r}-S_{\mathrm{ro}}}{1-S_{\mathrm{ro}}}$ is the effective degree of saturation which is defined based on $S_\mathrm{r}$ (water saturation) and residual degree of saturation $S_{\mathrm{ro}}$ (the water saturation at very high suction values which is mainly in the form of water films surrounding the particles). $\varTheta$ can be replaced by $S_\mathrm{r}$ for coarse-grained soils as a good approximation. In fact, $\alpha$ can be related to the inverse of air entry capillary pressure, while *n* is related to pore-size distribution. Parameter *m* is linked to *n* and is usually set to $\frac{n-1}{n}$. Tarantino (2009) considered the dependency of $\alpha$ (or air entry pressure) on stress level by relating it to the void ratio. He considered zero residual saturation for simplicity. Consequently, his modified formulation of van Genuchten equation reads (Tarantino 2009):

2 $$\begin{aligned} S_\mathrm{r}=\left( 1+\left( p_\mathrm{c}\left( \frac{e}{a}\right) ^\frac{1}{b}\right) ^n\right) ^{-b/n} \end{aligned}$$

where *e* is void ratio, *a*, *n* and *b* are fitting parameters. Recently, Salager et al. (2010, 2013) experimentally studied the void ratio dependency of retention curves and introduced the water retention surface, which clearly illustrates the change of saturation degree–capillary pressure relationship caused by a change in void ratio. Nuth and Laloui (2008) proposed a constitutive relationship between air entry value and void ratio as well as the elasto-plastic analogy in the degree of saturation versus capillary pressure relationship (an analogy with mechanical hysteresis) to model hysteretic water retention curves in deformable soils. By means of an empirical equation for the effective stress, Masin (2010) formulated and investigated the void ratio dependency of degree of saturation for various capillary pressure values. All these studies point to the fact that soil water retention curve changes as a result of a change in void ratio. Thus, stress-level dependency of the soil water retention curve has to be taken into account for a proper hydromechanical modelling. As indicated before, we expect not only to observe the stress-level dependency of the amount of different phases in unsaturated soils but also the stress measures have to be dependent on the amount of different phases. Such dependency has commonly been introduced to the current hydromechanical modelling frameworks using soil water retention (capillary pressure–saturation) curves.

There are different formulations for relating the state of stress in unsaturated soils to capillary pressure. Usually, this is done by defining an effective stress (Gallipoli et al. 2003a; Khalili et al. 2004; Gens et al. 2006; Sun et al. 2007, 2008). Among different stress state variables that have been proposed for modelling unsaturated soils, the effective stress has proved to work satisfactorily for modelling unsaturated soils (Khalili and Khabbaz 1998; Khalili et al. 2004; Lu 2008). It provides a consistent framework for both saturated and unsaturated conditions and is able to incorporate hydromechanical coupling. In most effective stress formulations, Bishop’s proposal for the effective stress equation (3) is employed along with different empirical equations for the effective stress parameter (Khalili et al. 2008; Uchaipichat 2010).

3 $$\begin{aligned} \varvec{\sigma }_{\mathrm{eff}}=\varvec{\sigma }-p_{\mathrm{air}}\varvec{I}+\chi p_\mathrm{c}\varvec{I} \end{aligned}$$

where $\varvec{\sigma }$ is the total stress tensor, $p_{\mathrm{air}}$ is the pore air pressure, $\chi$ is the so-called effective stress parameter, and $p_\mathrm{c}$ is the matric suction (capillary pressure) and $\varvec{I}$ denotes the unit tensor. In all such empirical equations, the effective stress parameter is explicitly expressed either in terms of the soil saturation or in terms of capillary pressure and parameters of soil water retention curve. For instance, Khalili and Khabbaz (1998) have introduced the following form for the effective stress parameter $\chi$:

4 $$\begin{aligned} \chi= & {} 1\quad \hbox {for}\,\frac{p_\mathrm{c}}{p_{\mathrm{c},\mathrm{ae}}} \le 1\nonumber \\ \chi= & {} \left( \frac{p_\mathrm{c}}{p_{\mathrm{c},\mathrm{ae}}}\right) ^\varOmega \quad \hbox {for}\, \frac{p_\mathrm{c}}{p_{\mathrm{c},\mathrm{ae}}}\ge 1 \end{aligned}$$

in which the $p_{\mathrm{c},\mathrm{ae}}$ is the air entry value and $\varOmega$ is a fitting parameter, which is suggested to be taken equal to 0.55 by Khalili and Khabbaz (1998) based on a large number of experimental data points they assessed.

A similar formula exists for the soil water retention curve which was proposed by Brooks and Corey (1964):

5 $$\begin{aligned} \varTheta= & {} 1\quad \hbox {for}\,\frac{p_\mathrm{c}}{p_{\mathrm{c},\mathrm{ae}}}\le 1\nonumber \\ \varTheta= & {} \left( \frac{p_\mathrm{c}}{p_{\mathrm{c},\mathrm{ae}}}\right) ^\lambda \quad \hbox {for}\,\frac{p_\mathrm{c}}{p_{\mathrm{c},\mathrm{ae}}}\ge 1 \end{aligned}$$

Comparison of Eqs. (4) and (5) indicates that the effective stress parameter and the effective degree of saturation are related. In fact, in the literature we find various equations for the effective stress parameter as a function of (or simply being equal to) effective degree of saturation (Lu et al. 2010). Vanapalli et al. (1996), for instance, expressed the contribution of the suction to shear strength of unsaturated soils $\tau _{\mathrm{us}}=\chi p_\mathrm{c} \hbox {tan}\,\phi ^{\prime }$, as follows:

6 $$\begin{aligned} \tau _{\mathrm{us}}=p_\mathrm{c}\varTheta ^\kappa \hbox {tan}\,\phi ^{\prime } \end{aligned}$$

where $\kappa$ is a material parameter and $\phi ^{\prime }$ is the effective friction angle, the same as the one for saturated soils. In some other cases, the effective stress parameter is expressed alternatively as a function of capillary pressure and air entry pressure as previously discussed (Khalili and Khabbaz 1998). From this brief review, we can conclude that the stress measures in unsaturated soils are closely connected to SWRC equation. But, a number of questions remain unanswered. One may ask how the stress-level dependency of soil water retention curve would be introduced to the current effective stress measures, which are empirically formulated. Moreover, it is not either known or proved how the coefficients appearing in the soil water retention curve are related to the empirical coefficients appearing in the proposed equations for the effective stress. The effect of net stress (stress level) on the soil water retention properties and consequently on the effective stress parameter has been mostly studied experimentally (Oh and Lu 2014) rather than through rigorous theoretical frameworks such as mixture theory. While some researchers such as Lu et al. (2010) and Nikooee et al. (2013) have resorted to an energetic and thermodynamic approach to formulate the effective stress in unsaturated soils, they have not looked into the stress-level dependency of hydraulic parameters directly and possible higher-order couplings it may bring about. A theoretical study is therefore essential to identify the scope of applicability of current formulations and to arrive at a most comprehensive formulation for the stress measures accounting for the all aspects of hydromechanical coupling.

In the current study, hence, we employ the mixture theory in order to study the mutual relationship between SWRC equation and a stress measure for an unsaturated porous medium. In fact, in this study we are limited to current hydraulic formulations that account for the hydraulic hysteresis (different equations for drying, wetting and scanning branches need to be introduced). Also, the contribution of interfaces to the mechanical behaviour of the unsaturated porous medium has been neglected. This can be a reasonable assumption when coarse-grained soils are considered. In fine-grained soils especially in scanning curves and/or in low saturation degrees, the contribution of interfaces can be considerable (Nikooee et al. 2013; Likos 2014; Gray and Schrefler 2001; Dangla and Coussy 2002). In what follows, first the theoretical framework is introduced. Then, the expected coupling is derived based on the definition of the strain energy function of the mixture. Next, coupling/connection between the stress measure and soil water retention curve is discussed and compared to current formulations. Finally, concluding remarks and some suggestions for future studies are provided.

## Basic Assumptions

We consider three phases: a solid (superscript s), a wetting fluid (superscript w) and a non-wetting fluid (superscript nw). The solid deforms from an initial configuration $\varvec{X}$ to a current configuration $\varvec{x}$. The deformation gradient tensor of the solid

7 $$\begin{aligned} \varvec{F}=\frac{\textstyle \partial \varvec{x}}{\textstyle \partial \varvec{X}}^\mathrm{T} \end{aligned}$$

In Eq. (7), the usual superscript s is left out because in the subsequent equations we do not consider deformation gradient tensors of other constituents. The determinant of the deformation gradient tensor represents the volume change of the mixture

8 $$\begin{aligned} J=\mathrm {det}\,\varvec{F}\end{aligned}$$

## Conservation Laws and Constitutive Laws

Excluding mass transfer between phases, the mass balance of each phase is then written as:

9 $$\begin{aligned} \frac{\textstyle \partial \rho ^\alpha }{\textstyle \partial t} + \varvec{\nabla }\cdot \left( \rho ^\alpha \varvec{v}^\alpha \right) = 0 , \quad \alpha = \hbox {s}, \hbox {w}, \hbox {nw} \end{aligned}$$

in which $\rho ^\alpha$ is the apparent density and $\varvec{v}^\alpha$ the velocity of component $\alpha$. In analogy to Vankan et al. (1996), Huyghe and Janssen (1997) and Huyghe and Bovendeerd (2004), we refer current descriptors of the mixture with respect to an initial state of the porous solid. If we introduce the Lagrangian apparent density [compare to eq. (4.1), in Bowen (1980)]

10 $$\begin{aligned} R^\alpha =J \rho ^\alpha \end{aligned}$$

per unit initial volume of the mixture, we can rewrite the mass balance equation (9) as follows:

11 $$\begin{aligned} \frac{D^\mathrm{s} R^\alpha }{Dt}+J\varvec{\nabla }\cdot \left[ \rho ^\alpha \left( \varvec{v}^\alpha -\varvec{v}^\mathrm{s}\right) \right] =0 \end{aligned}$$

In order to simplify our task, we assume the solid and the wetting phase (usually water) to be incompressible, so that the intrinsic density

12 $$\begin{aligned} \rho ^\alpha _i=\frac{\rho ^\alpha }{\phi ^\alpha } \quad \alpha =\hbox {s},\hbox {w} \end{aligned}$$

is constant. $\phi ^\alpha$ is the volume fraction of phase $\alpha$. For the non-wetting phase (often air), we assume the phase to be drained, i.e. the pressure of the air is atmospheric everywhere. Because of the constant pressure and the assumption of isotherm, the intrinsic density

13 $$\begin{aligned} \rho ^{\mathrm{nw}}_i=\frac{\rho ^{\mathrm{nw}}}{\phi ^{\mathrm{nw}}} \end{aligned}$$

of the air is constant in time and space. Because of Eqs. (12) and (13), the mass conservation equations reduce to volume conservation equations:

14 $$\begin{aligned} \frac{D^\mathrm{s} \varPhi ^\alpha }{Dt}+J\varvec{\nabla }\cdot \left[ \phi ^\alpha \left( \varvec{v}^\alpha -\varvec{v}^\mathrm{s}\right) \right] =0 \end{aligned}$$

in which

15 $$\begin{aligned} \varPhi ^\alpha =J\phi ^\alpha \end{aligned}$$

and $\frac{D^\alpha }{Dt}$ is the time derivative for an observer fixed to the constituent $\alpha$. The total mass balance is obtained by adding the three mass balances together:

16 $$\begin{aligned} -J\sum _{\beta = \mathrm{w},\mathrm{nw}}\varvec{\nabla }\cdot \left[ \phi ^\beta \left( \varvec{v}^\beta -\varvec{v}^\mathrm{s}\right) \right] =J\varvec{\nabla }\cdot \varvec{v}^\mathrm{s} \end{aligned}$$

Neglecting body forces and inertia, the momentum balance takes the form:

17 $$\begin{aligned} \varvec{\nabla }\cdot \varvec{\sigma }^\alpha + \varvec{\pi }^\alpha = \mathbf{0}, \quad \alpha = \mathrm{s}, \mathrm{w}, \mathrm{nw} \end{aligned}$$

in which $\varvec{\sigma }^\alpha$ is the partial stress tensor of constituent $\alpha$ and $\varvec{\pi }^\alpha$ is the momentum imparted upon phase $\alpha$ by the other phases, which after summation over the three components yields:

18 $$\begin{aligned} \varvec{\nabla }\cdot \varvec{\sigma }=\varvec{\nabla }\cdot \varvec{\sigma }^\mathrm{s} + \varvec{\nabla }\cdot \varvec{\sigma }^\mathrm{w} + \varvec{\nabla }\cdot \varvec{\sigma }^{\mathrm{nw}} = \mathbf{0} \end{aligned}$$

if use is made of the balance condition:

19 $$\begin{aligned} \varvec{\pi }^\mathrm{s} + \varvec{\pi }^\mathrm{w} + \varvec{\pi }^{\mathrm{nw}}= \mathbf{0} \end{aligned}$$

Although the non-wetting phase is drained and non-wetting pressure is atmospheric pressure, the momentum interaction $\varvec{\pi }^{\mathrm{nw}}$ is nonzero if gradients of non-wetting volume fraction exist. However, the gradient in non-wetting volume fraction is a self-equilibrated term that does not induce flow. Balance of moment of momentum requires that the stress tensor $\varvec{\sigma }$ be symmetric. If no moment of momentum interaction between components occurs, the partial stresses $\varvec{\sigma }^\alpha$ also are symmetric. In this paper, we assume all partial stresses to be symmetric. Under isothermal conditions, the entropy inequality for a unit volume of mixture reads Bowen (1980):

20 $$\begin{aligned} \sum _{\alpha = \mathrm{s},\mathrm{w},\mathrm{nw}}^{} \left( -\rho ^\alpha \frac{D^\alpha F^\alpha }{Dt} + \varvec{\sigma }^\alpha \mathbf{{\ :\ }}\varvec{D}^\alpha - \varvec{\pi }^\alpha \cdot \varvec{v}^\alpha \right) \ge 0. \end{aligned}$$

in which $F^\alpha$ is the Helmholtz free energy of constituent $\alpha$ per unit mass constituent. We introduce the strain energy function [compare to Biot (1972, eq. (3.22)) and Bowen (1980, eq. (4.2))]

21 $$\begin{aligned} W=J\sum _{\alpha = \mathrm{s},\mathrm{w},\mathrm{nw}}^{} \rho ^\alpha F^\alpha =J\sum _{\alpha = \mathrm{s},\mathrm{w},\mathrm{nw}}^{} \psi ^\alpha \end{aligned}$$

as the Helmholtz free energy of a mixture volume which in the *initial* state of the solid equals unity. $\psi ^\alpha$ is the Helmholtz free energy of constituent $\alpha$ per unit mixture volume. Rewriting the inequality (20) for the entropy production per initial mixture volume—i.e. we multiply inequality (20) by the relative volume change *J*—we find:

22 $$\begin{aligned}&-\frac{D^\mathrm{s}}{Dt} W + J \varvec{\sigma }\mathbf{{\ :\ }}\varvec{\nabla }\varvec{v}^\mathrm{s} +\,J\varvec{\nabla }\cdot \left[ \left( \varvec{v}^\mathrm{w}-\varvec{v}^\mathrm{s}\right) \cdot \varvec{\sigma }^\mathrm{w} - \left( \varvec{v}^\mathrm{w} - \varvec{v}^\mathrm{s}\right) \psi ^\mathrm{w}\right] \nonumber \\&\quad +J\varvec{\nabla }\cdot \left[ \left( \varvec{v}^{\mathrm{nw}}-\varvec{v}^\mathrm{s}\right) \cdot \varvec{\sigma }^{\mathrm{nw}} - \left( \varvec{v}^{\mathrm{nw}} - \varvec{v}^\mathrm{s}\right) \psi ^{\mathrm{nw}}\right] \ge 0. \end{aligned}$$

The entropy inequality should hold for an arbitrary state of the mixture, complying with the balance laws. We introduce into the entropy inequality (22) the total mass balance (16) using a Lagrange multiplier *p*:

23 $$\begin{aligned}&-\frac{D^\mathrm{s}}{Dt} W + J \varvec{\sigma }_{\mathrm{eff}}\mathbf{{\ :\ }}\varvec{\nabla }\varvec{v}^\mathrm{s}+ J\sum _{\beta = \mathrm{w},\mathrm{nw}}\left( \varvec{\sigma }^\beta +\left( p\phi ^\beta -\psi ^\beta \right) \varvec{I}\right) :\varvec{\nabla }\left( \varvec{v}^\beta -\varvec{v}^\mathrm{s}\right) \nonumber \\&\quad \quad +J\sum _{\beta = w,nw}\left( \varvec{v}^\beta - \varvec{v}^\mathrm{s}\right) \cdot \left( -\varvec{\nabla }\psi ^\beta +p\varvec{\nabla }\phi ^\beta +\varvec{\nabla }\cdot \varvec{\sigma }^\beta \right) \ge 0. \end{aligned}$$

with $\varvec{\sigma }_{\mathrm{eff}}$ the effective stress:

24 $$\begin{aligned} \varvec{\sigma }_{\mathrm{eff}}=\varvec{\sigma }+p\varvec{I} \end{aligned}$$

The Lagrange multiplier *p* has obviously the dimension of a pressure. We choose as independent variables the Green strain $\varvec{E}$, the Lagrangian form of the volume fractions of the fluid and air $\varPhi ^\mathrm{w}$ and $\varPhi ^{\mathrm{nw}}$, and of the relative velocity $\varvec{v}^{\mathrm{ws}}=\varvec{F}^{-1}\cdot (\varvec{v}^\mathrm{w} - \varvec{v}^\mathrm{s})$ and $\varvec{v}^{\mathrm{nws}}=\varvec{F}^{-1}\cdot (\varvec{v}^{\mathrm{nw}} - \varvec{v}^\mathrm{s})$. We apply the principle of equipresence, i.e. all dependent variables depend on all independent variables, unless the entropy inequality requires otherwise:

25 $$\begin{aligned} W= & {} W\left( \varvec{E},\varPhi ^\mathrm{w},\varPhi ^\mathrm{nw},\varvec{v}^\mathrm{ws},\varvec{v}^\mathrm{nws}\right) \end{aligned}$$

26 $$\begin{aligned} \varvec{\sigma }= & {} \varvec{F}\cdot \varvec{\sigma }_*\left( \varvec{E},\varPhi ^\mathrm{w},\varPhi ^\mathrm{nw},\varvec{v}^\mathrm{ws},\varvec{v}^\mathrm{nws}\right) \cdot \varvec{F}^{\mathrm{C}}\end{aligned}$$

27 $$\begin{aligned} \varvec{\sigma }^\mathrm{w}= & {} \varvec{F}\cdot \varvec{\sigma }^\mathrm{w}_*\left( \varvec{E},\varPhi ^\mathrm{w},\varPhi ^\mathrm{nw},\varvec{v}^\mathrm{ws},\varvec{v}^\mathrm{nws}\right) \cdot \varvec{F}^{\mathrm{C}}\end{aligned}$$

28 $$\begin{aligned} \varvec{\sigma }^\mathrm{nw}= & {} \varvec{F}\cdot \varvec{\sigma }^\mathrm{nw}_*\left( \varvec{E},\varPhi ^\mathrm{w},\varPhi ^\mathrm{nw},\varvec{v}^\mathrm{ws},\varvec{v}^\mathrm{nws}\right) \cdot \varvec{F}^{\mathrm{C}}\end{aligned}$$

29 $$\begin{aligned} \varvec{\pi }^\mathrm{w}= & {} \varvec{F}\cdot \varvec{\pi }^\mathrm{w}_*\left( \varvec{E},\varPhi ^\mathrm{w},\varPhi ^\mathrm{nw},\varvec{v}^\mathrm{ws},\varvec{v}^\mathrm{nws}\right) \end{aligned}$$

30 $$\begin{aligned} \varvec{\pi }^\mathrm{nw}= & {} \varvec{F}\cdot \varvec{\pi }^\mathrm{nw}_*\left( \varvec{E},\varPhi ^\mathrm{w},\varPhi ^\mathrm{nw},\varvec{v}^\mathrm{ws},\varvec{v}^\mathrm{nws}\right) \end{aligned}$$

We apply the chain rule for time differentiation of *W*:

31 $$\begin{aligned}&\left( J \varvec{\sigma }_{\mathrm{eff}} - \varvec{F}\cdot \frac{\textstyle \partial \textstyle W}{\textstyle \partial \textstyle \varvec{E}}\cdot \varvec{F}^\mathrm{T}\right) \mathbf{{\ :\ }}\varvec{\nabla }\varvec{v}^\mathrm{s} + -\frac{\textstyle \partial \textstyle W}{\textstyle \partial \textstyle \varvec{v}^{\mathrm{ws}}} \cdot \frac{D^\mathrm{s}}{Dt}\varvec{v}^{\mathrm{ws}}-\frac{\textstyle \partial \textstyle W}{\textstyle \partial \textstyle \varvec{v}^{\mathrm{nws}}} \cdot \frac{D^\mathrm{s}}{Dt}\varvec{v}^{\mathrm{nws}}\nonumber \\&\quad +J\left[ \varvec{\sigma }^\mathrm{w}+\left( \mu ^\mathrm{w} \phi ^\mathrm{w}-\psi ^\mathrm{w}\right) \varvec{I}\right] \mathbf{{\ :\ }}\varvec{\nabla }\left( \varvec{v}^\mathrm{w}-\varvec{v}^\mathrm{s}\right) \nonumber \\&\quad +J\left[ \varvec{\sigma }^{\mathrm{nw}}+\left( \mu ^{\mathrm{nw}} \phi ^{\mathrm{nw}}-\psi ^{\mathrm{nw}}\right) \varvec{I}\right] \mathbf{{\ :\ }}\varvec{\nabla }\left( \varvec{v}^{\mathrm{nw}}-\varvec{v}^\mathrm{s}\right) \nonumber \\&\quad +J\sum _{\beta =\mathrm{w},\mathrm{nw}}\left( \varvec{v}^\beta -\varvec{v}^\mathrm{s}\right) \cdot \left( -\varvec{\nabla }\psi ^\beta +\mu ^\beta \varvec{\nabla }\phi ^\beta + \varvec{\nabla }\cdot \varvec{\sigma }^\beta \right) \ge 0. \end{aligned}$$

in which $\mu ^\mathrm{w}$ is the chemical potential of the wetting fluid:

32 $$\begin{aligned} \mu ^\mathrm{w}=p+\frac{\textstyle \partial W}{\textstyle \partial \varPhi ^\mathrm{w}} \end{aligned}$$

and $\mu ^{\mathrm{nw}}$ is the chemical potential of the non-wetting phase:

33 $$\begin{aligned} \mu ^{\mathrm{nw}}=p+\frac{\textstyle \partial W}{\textstyle \partial \varPhi ^{\mathrm{nw}}} \end{aligned}$$

Equation (31) should be true for any value of the state variables. Close inspection of the choice of independent variables and the inequality (31), reveals that the first term of (31) is linear in the solid velocity gradient $\varvec{\nabla }\varvec{v}^s$, the second term linear in $\frac{D^\mathrm{s}}{Dt}\varvec{v}^{\mathrm{ws}}$, the third term linear in $\frac{D^\mathrm{s}}{Dt}\varvec{v}^{\mathrm{nws}}$, the fourth term linear in the relative velocity gradients $\varvec{\nabla }(\varvec{v}^\mathrm{w}-\varvec{v}^\mathrm{s})$ and the fifth term linear in the relative velocity gradients $\varvec{\nabla }(\varvec{v}^{\mathrm{nw}}-\varvec{v}^\mathrm{s})$. Therefore, by a standard argument, we find:

34 $$\begin{aligned}&\displaystyle \varvec{\sigma }_{\mathrm{eff}} = \frac{1}{J} \varvec{F}\cdot \frac{\textstyle \partial \textstyle W}{\textstyle \partial \textstyle \varvec{E}}\cdot \varvec{F}^\mathrm{T} \end{aligned}$$

35 $$\begin{aligned}&\displaystyle \frac{\textstyle \partial \textstyle W}{\textstyle \partial \textstyle \varvec{v}^{\mathrm{ws}}}=\mathbf{0}\end{aligned}$$

36 $$\begin{aligned}&\displaystyle \frac{\textstyle \partial \textstyle W}{\textstyle \partial \textstyle \varvec{v}^{\mathrm{nws}}}=\mathbf{0}\end{aligned}$$

37 $$\begin{aligned}&\displaystyle \varvec{\sigma }^\mathrm{w}=\left( \psi ^\mathrm{w}-\mu ^\mathrm{w} \phi ^w\right) \varvec{I}\end{aligned}$$

38 $$\begin{aligned}&\displaystyle \varvec{\sigma }^{\mathrm{nw}}=\left( \psi ^{\mathrm{nw}}-\mu ^{\mathrm{nw}} \phi ^{\mathrm{nw}}\right) \varvec{I}\end{aligned}$$

leaving as inequality:

39 $$\begin{aligned} J\sum _{\beta =\mathrm{w},\mathrm{nw}}\left( \varvec{v}^\beta -\varvec{v}^\mathrm{s}\right) \cdot \left( -\varvec{\nabla }\psi ^\beta +\mu ^\beta \varvec{\nabla }\phi ^\beta + \varvec{\nabla }\cdot \varvec{\sigma }^\beta \right) \ge 0. \end{aligned}$$

Equation (34) indicates that the effective stress of the mixture can be derived from a strain energy function W which represents the free energy of the mixture. From Eqs. 24 and 34, one can infer that the pressure *p* can be interpreted as the pressure present at the interface between solid and wetting fluid. Indeed, the stress in the solid has a hydrostatic component that does not induce deformation of the incompressible solid. This is the pressure exerted on the solid by the wetting fluid. Equation (34) is the constitutive law of the solid skeleton, which unlike the solid itself is compressible as its porosity can change during deformation. Equations (35) and (36) show that the strain energy function cannot depend on the relative velocities. Thus, the stress of an unsaturated medium can be derived from a regular strain energy function, which physically has the same meaning as in solid materials, but which can depend on both strain and composition of the medium. According to Eqs. (37) and (38), the partial stress of the fluid and the gas is a scalar. Transforming the relative velocity to its Lagrangian equivalent, we find instead of (39):

40 $$\begin{aligned} \sum _{\beta =\mathrm{w},\mathrm{nw}}\varvec{v}^{\beta \mathrm{s}}\cdot \left[ -\varvec{\nabla }_0\psi ^\beta +\mu ^ \beta \varvec{\nabla }_0 \phi ^\beta +\varvec{\nabla }_0\cdot \varvec{\sigma }^\beta \right] \ge 0. \end{aligned}$$

in which $\varvec{\nabla }_0=\varvec{F}^\mathrm{T}\cdot \varvec{\nabla }$ is the gradient operator with respect to the initial configuration. Because Reynolds numbers are usually very low, we assume that the system is not too far from equilibrium. Hence, we can express the dissipation (40) associated with relative flow of fluid as a quadratic function of the relative velocities:

41 $$\begin{aligned} -\varvec{\nabla }_0\psi ^\beta +\mu ^\beta \varvec{\nabla }_0 \phi ^\beta +\varvec{\nabla }_0\cdot \varvec{\sigma }^\beta =\sum _{\gamma =\mathrm{w},\mathrm{nw}}\varvec{B}^{\beta \gamma }\cdot \varvec{v}^{\gamma s} \end{aligned}$$

$\varvec{B}^{\beta \gamma }$ is a positive definite matrix of frictional coefficients. Substituting Eq. (37) into Eq. (41) yields Lagrangian forms of the classical equations of irreversible thermodynamics:

42 $$\begin{aligned} -\phi ^\mathrm{w}\varvec{\nabla }_0\mu ^\beta =\sum _{\gamma =\mathrm{w},\mathrm{nw}}\varvec{B}^{\beta \gamma }\cdot \varvec{v}^{\gamma \mathrm{s}} \end{aligned}$$

## Corey–Brooks Relationship for Capillary Pressure and Bishop’s Equation

The pores of the incompressible solid are filled with an incompressible wetting fluid and air at atmospheric pressure. The sample as a whole can change its volume by expelling wetting or non-wetting fluid. The sample is in contact with a water reservoir at zero pressure. At the interface between the reservoir and the sample, the chemical potential $\mu ^\mathrm{w}$ of the wetting phase is the same inside and outside:

43 $$\begin{aligned} \mu ^\mathrm{w}=p+\frac{\textstyle \partial W}{\textstyle \partial \varPhi ^\mathrm{w}}=\mu ^\mathrm{w}_{\mathrm{ext}} \end{aligned}$$

the chemical potential of the non-wetting phase is:

44 $$\begin{aligned} \mu ^{\mathrm{nw}}=p+\frac{\textstyle \partial W}{\textstyle \partial \varPhi ^{\mathrm{nw}}}=\mu ^{\mathrm{nw}}_{\mathrm{ext}}=\frac{RT}{{\overline{V}}_{\mathrm{air}}} \mathrm {ln}\frac{p_{\mathrm{air}}}{p_0}\approx p_{\mathrm{air}}-p_0 \end{aligned}$$

The capillary pressure difference between the wetting fluid and non-wetting fluid is [Bowen 1980, p. 1141, eq. (5.20)]:

45 $$\begin{aligned} p_\mathrm{c}=-\frac{\partial W}{\partial \varPhi ^\mathrm{w}} \end{aligned}$$

in which $p_\mathrm{c}$ is the capillary pressure of the wetting fluid and $\varPhi ^\mathrm{w}$ the Lagrangian volume fraction of the wetting fluid:

46 $$\begin{aligned} \varPhi ^\mathrm{w}=J\phi ^\mathrm{w}=\frac{J\rho ^\mathrm{w}}{\rho ^\mathrm{w}_i}=\frac{R^\mathrm{w}}{\rho ^\mathrm{w}_i} \end{aligned}$$

The saturation is defined as:

47 $$\begin{aligned} S=\frac{\phi ^\mathrm{w}}{1-\phi ^\mathrm{s}}=\frac{\varPhi ^\mathrm{w}}{\left( J-\phi ^\mathrm{s}_0\right) } \end{aligned}$$

According to Gallipoli (2012), Brooks–Corey can be written for deformable media as,

48 $$\begin{aligned} S=\left( \frac{p_\mathrm{c}}{p_{\mathrm{c},\mathrm{ae}}}\right) ^{-\lambda } \end{aligned}$$

for

49 $$\begin{aligned} p_{\mathrm{c}} \ge p_{\mathrm{c},\mathrm{ae}} \end{aligned}$$

in which we assume that the effective saturation is equal to the saturation and $p_{\mathrm{c},\mathrm{ae}}$ is the air entry pressure dependent on the void ratio

50 $$\begin{aligned} e=\frac{1-\phi ^\mathrm{s}}{\phi ^\mathrm{s}}=\frac{J-\phi ^\mathrm{s}_0}{\phi ^\mathrm{s}_0} \end{aligned}$$

according to

51 $$\begin{aligned} p_{\mathrm{c},\mathrm{ae}}=\frac{\kappa }{\hbox {e}^\xi } \end{aligned}$$

yielding a water retention curve for deformable media:

52 $$\begin{aligned} S=\left( \frac{\hbox {e}^\xi p_\mathrm{c}}{\kappa }\right) ^{-\lambda } \end{aligned}$$

Inverting this relationship yields

53 $$\begin{aligned} p_\mathrm{c}=\kappa \hbox {e}^{-\xi }S^{\frac{-1}{\lambda }} \end{aligned}$$

or, after substitution into Eq. (45):

54 $$\begin{aligned} -\frac{\partial W}{\partial \varPhi ^\mathrm{w}}=\kappa \hbox {e}^{-\xi }S^{\frac{-1}{\lambda }} \end{aligned}$$

or

55 $$\begin{aligned} -\frac{\partial W}{\partial \varPhi ^\mathrm{w}}=\kappa \left( \frac{J-\phi ^\mathrm{s}_0}{\phi ^\mathrm{s}_0}\right) ^{-\xi }S^{\frac{-1}{\lambda }} \end{aligned}$$

Differentiating the above equation with respect to strain yields:

56 $$\begin{aligned} -\frac{\partial \left( J\varvec{F}^{-1}\cdot \varvec{\sigma }_{\mathrm{eff}}\cdot \varvec{F}^{-\mathrm{C}}\right) }{\partial \varPhi ^\mathrm{w}}= -\frac{\kappa \xi }{\phi ^\mathrm{s}_0}\left( \frac{J-\phi ^\mathrm{s}_0}{\phi ^\mathrm{s}_0}\right) ^{-\xi -1}S^{\frac{-1}{\lambda }}\frac{\hbox {d}J}{\hbox {d}\varvec{E}} \end{aligned}$$

in which

57 $$\begin{aligned} \frac{\hbox {d}J}{d\varvec{E}}=2\frac{\hbox {d}J}{\hbox {d}\left( \varvec{F}^\mathrm{T}\cdot \varvec{F}\right) }=J\varvec{F}^{-1}\cdot \varvec{F}^{-\mathrm{C}} \end{aligned}$$

In Eq. (57), we use the identity

58 $$\begin{aligned} \frac{\hbox {d} \mathrm {det}\varvec{A}}{\hbox {d}\varvec{A}}=\mathrm {det}(\varvec{A})\varvec{A}^{-1} \end{aligned}$$

for any tensor $\varvec{A}$. This results in the following expression for the stress:

59 $$\begin{aligned} \varvec{\sigma }_{\mathrm{eff}}= & {} \varvec{\sigma }_{\mathrm{sat}}+\int _{J-\phi ^\mathrm{s}_0}^{\varPhi ^\mathrm{w}}\frac{\partial p_\mathrm{c}}{\partial \varvec{E}}\hbox {d}\Phi ^\mathrm{w} \end{aligned}$$

60 $$\begin{aligned}= & {} \varvec{\sigma }_{\mathrm{sat}}+\int _{J-\phi ^\mathrm{s}_0}^{\varPhi ^\mathrm{w}}\left[ \frac{\kappa \xi }{\phi ^s_0}\left( \frac{J-\phi ^\mathrm{s}_0}{\phi ^\mathrm{s}_0}\right) ^{-\xi -1}S^{\frac{-1}{\lambda }}\right] \hbox {d}\varPhi ^\mathrm{w} \varvec{I} \end{aligned}$$

in which $\varvec{\sigma }_{\mathrm{sat}}$ is an integration constant dependent on the strain $\varvec{E}$, representing the effective stress in the saturated case. Equation (60) is rewritten in the form:

61 $$\begin{aligned} \varvec{\sigma }_{\mathrm{eff}}=\varvec{\sigma }_{\mathrm{sat}}+\int _1^{\mathrm{S}}\left( J-\phi ^\mathrm{s}_0\right) \left[ \frac{\kappa \xi }{\phi ^s_0}\left( \frac{J-\phi ^\mathrm{s}_0}{\phi ^\mathrm{s}_0}\right) ^{-\xi -1}S^{\frac{-1}{\lambda }}\right] \hbox {d}S\varvec{I} \end{aligned}$$

or, after integration:

62 $$\begin{aligned} \varvec{\sigma }_{\mathrm{eff}}= & {} \varvec{\sigma }_{\mathrm{sat}}+\left( J-\phi ^\mathrm{s}_0\right) \left[ \frac{\kappa \xi }{\phi ^\mathrm{s}_0\left( \frac{-1}{\lambda }+1\right) }\left( \frac{J-\phi ^s_0}{\phi ^s_0}\right) ^{-\xi -1}S^{\frac{-1}{\lambda }+1}\right] \varvec{I}|_1^{\mathrm{S}}\nonumber \\= & {} \varvec{\sigma }_{\mathrm{sat}}+\left[ \frac{\kappa \xi }{\frac{-1}{\lambda }+1}\left( \frac{J-\phi ^\mathrm{s}_0}{\phi ^\mathrm{s}_0}\right) ^{-\xi }\left( S^{\frac{-1}{\lambda }+1}-1\right) \right] \varvec{I} \end{aligned}$$

Considering that

63 $$\begin{aligned} \varvec{\sigma }_{\mathrm{sat}}=\varvec{\sigma }- p_{\mathrm{c},\mathrm{ae}}\varvec{I}\end{aligned}$$

we can rewrite Eq. (62)

64 $$\begin{aligned} \varvec{\sigma }_{\mathrm{eff}}=\sigma -p_{\mathrm{c},\mathrm{ae}}\varvec{I}+\left[ \frac{\kappa \lambda \xi }{\lambda -1}\left( \frac{J-\phi ^s_0}{\phi ^s_0}\right) ^{-\xi }\left( S^{\frac{-1}{\lambda }+1}-1\right) \right] \varvec{I}\end{aligned}$$

Making use of Eq. (50)

65 $$\begin{aligned} \varvec{\sigma }_{\mathrm{eff}}=\sigma -p_{\mathrm{c},\mathrm{ae}}\varvec{I}+\left[ \frac{\kappa \lambda \xi }{\lambda -1}\hbox {e}^{-\xi }\left( S^{\frac{-1}{\lambda }+1}-1\right) \right] \varvec{I}\end{aligned}$$

Applying Eqs. (51) and (53)

66 $$\begin{aligned} \varvec{\sigma }_{\mathrm{eff}}= & {} \sigma -p_\mathrm{c}\left( \frac{p_{\mathrm{c},\mathrm{ae}}}{p_\mathrm{c}}\left( 1+\frac{\lambda \xi }{\lambda -1}\right) -\left( \frac{p_{\mathrm{c}}}{p_{\mathrm{c},\mathrm{ae}}}\right) ^{-\lambda }\left( \frac{\lambda \xi }{\lambda -1}\right) \right) \varvec{I}\end{aligned}$$

67 $$\begin{aligned} \chi= & {} \left( \frac{p_{\mathrm{c},\mathrm{ae}}}{p_\mathrm{c}}\left( 1+\frac{\lambda \xi }{\lambda -1}\right) -\left( \frac{p_{\mathrm{c},\mathrm{ae}}}{p_\mathrm{c}}\right) ^{\lambda }\left( \frac{\lambda \xi }{\lambda -1}\right) \right) \end{aligned}$$

This is a general form of Khalili and Khabbaz (1998) who found the effective stress parameter to be a function of $\frac{p_{\mathrm{c}}}{p_\mathrm{c},\mathrm{ae}}$

## Results and Discussion

**Fig. 1:**
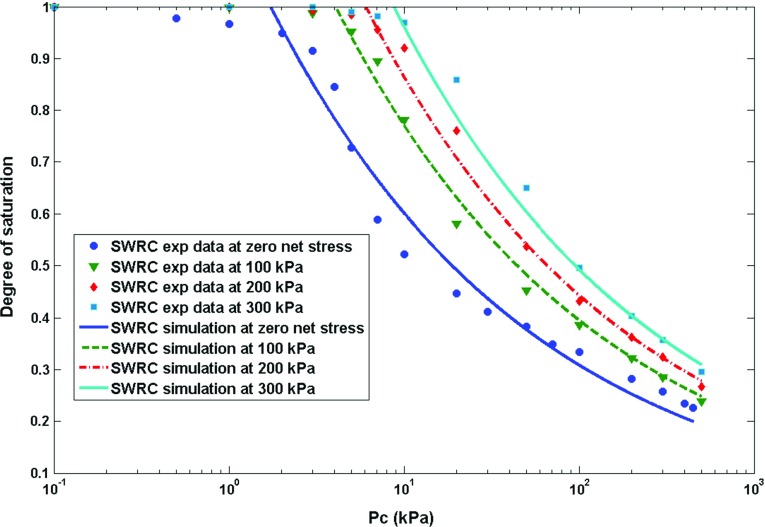
Soil water retention curve at different net stress levels (weathered granite), experimental data are from Lee et al. (2005)

**Fig. 2:**
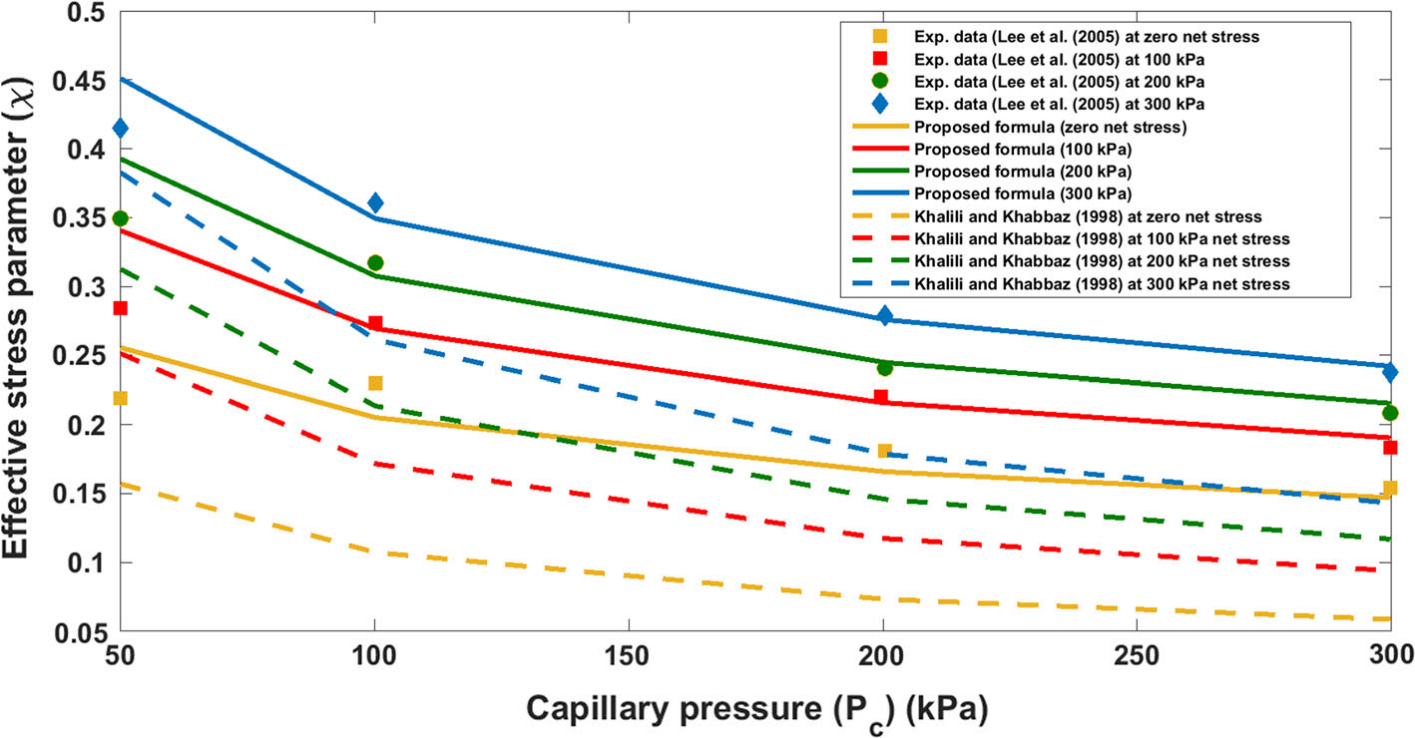
Effective stress parameter versus suction at different net stress levels: comparing the proposed equation and the experimental data (illustration for weathered granite)

**Fig. 3:**
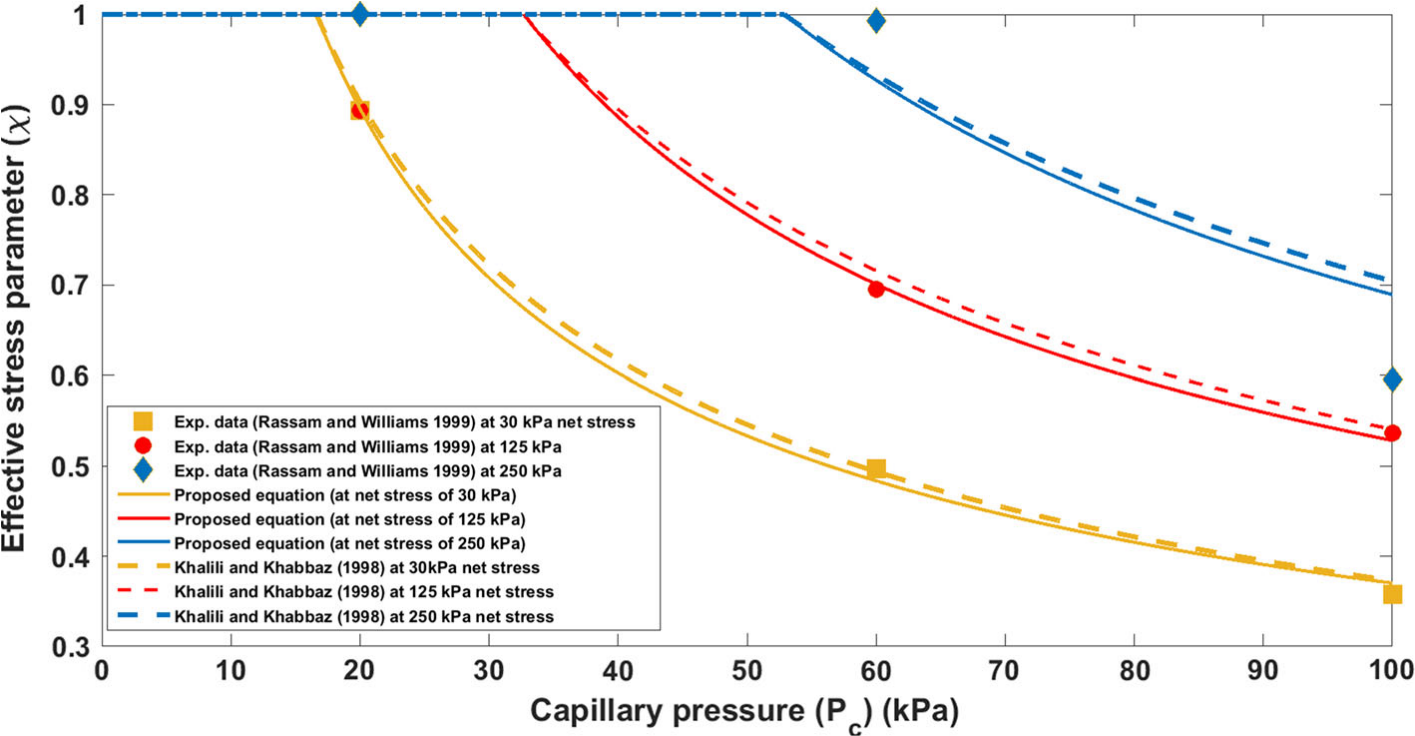
Effective stress parameter versus suction at different net stress levels: comparing the proposed equation and the experimental data (illustration for Kidston tailing)

**Fig. 4:**
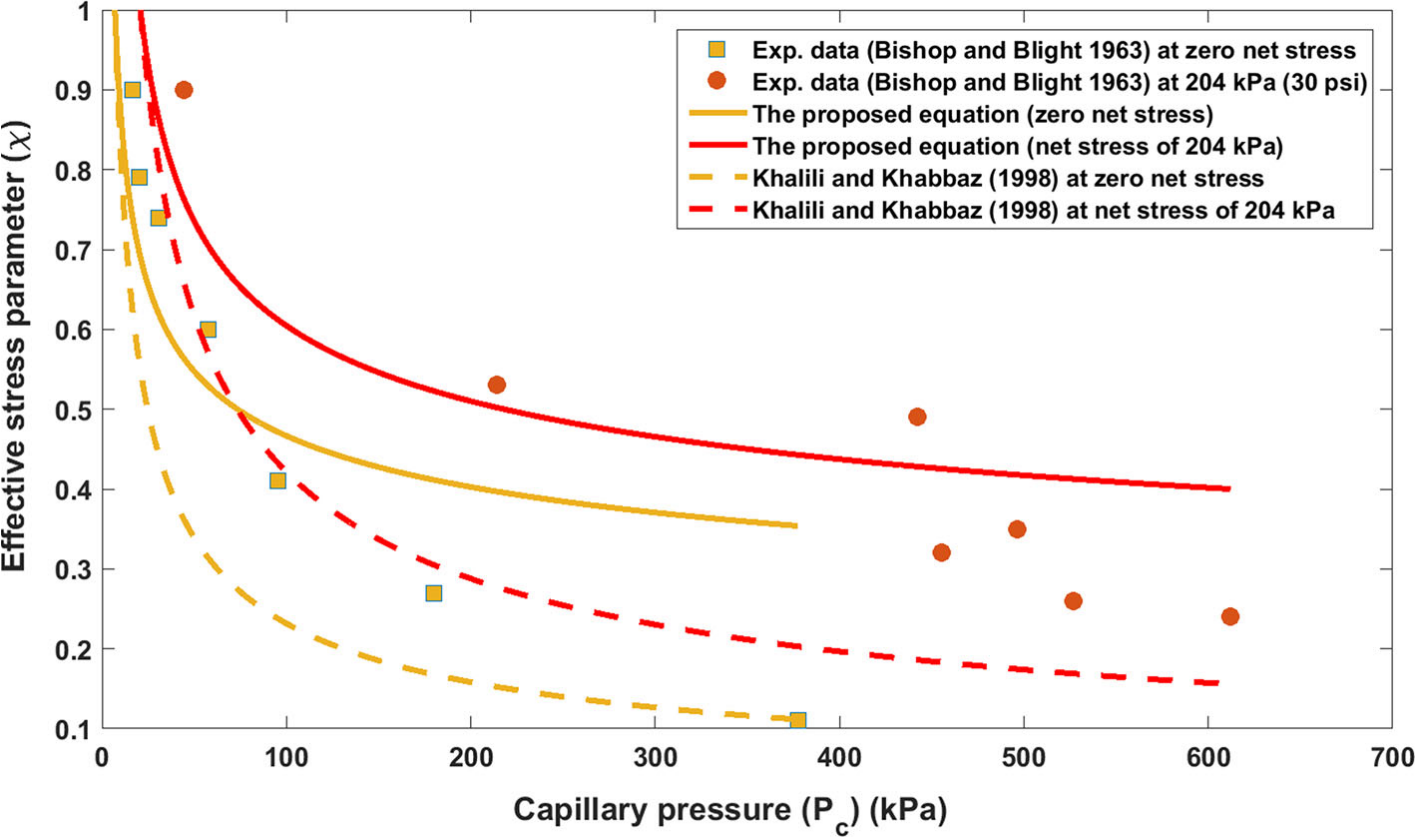
Effective stress parameter versus suction at different net stress levels: comparing the proposed equation and the experimental data (illustration for Talybont clay)

It has been shown that the drainage imbibition curve of a deformable sample is inextricably linked to the state of deformation of the sample. In case of an incompressible solid and incompressible wetting fluid, this interdependence has been derived from the second law of thermodynamics and is given by Eq. (60). As it was elaborated in the previous section, this relationship leads to the formulation of the effective stress for a deforming unsaturated medium. In order to evaluate the efficacy and accuracy of the proposed equation, experimental values of the effective stress parameter at different net stress and suction values extracted from experimental data of unsaturated shear strength behaviour of weathered granite (Lee et al. 2005), Kidston tailing (Rassam and Williams 1999) and Talybont clay (Bishop and Blight 1963) were employed. The merit of the proposed equation is that it is based on a rigorous thermodynamic framework, and therefore, it results in formulating the effective stress parameter based on the physically meaningful parameters. The parameters that are required in order to estimate the effective stress parameter at different net stress and capillary pressure levels are basically the parameters which describe the equation of the soil water retention curve at different net stress levels. Furthermore, we compared our proposed equation to that of Khalili and Khabbaz (1998).In order to improve the predictive capability of the equation proposed by Khalili and Khabbaz (1998), we use the air entry value of the unsaturated soils samples associated with each net stress level (the same as what we consider for our proposed equation), and for the exponent of their equation, we keep it constant and equal to their suggested best fit value of 0.55. This will make $\varOmega$ a material independent parameter (this is in line with the assumption of previous researchers, e.g. that of Masin (2010), who investigated the void ratio dependency of soil water retention curves using an effective stress approach). Lee et al. (2005) made a novel device to determine the soil water retention curve at different net stress levels. However, the current experimental practice in unsaturated soil mechanics is solely to determine the soil water retention curve using plate pressure apparatus at zero net stress level. Of course, the water retention properties at different net stress levels can also be obtained from the triaxial testing apparatus, but the measurement of the water volume change in this device is not as accurate as plate pressure apparatus, and therefore, there are limited data in the literature on the soil water retention properties at different net stress levels. Hereafter, in order to explore different experimental datasets in the literature, we present a systematic approach to use the proposed formula for the determination of the effective stress parameter at different net stress and suction values. For weathered granite, the experimental data on the water retention curve at four net stress levels, namely 0, 100, 200, and 300 kPa, are available (Fig. 1). Therefore, first using the effective parameter values at different suction levels, and at zero net stress the value of coupling parameter is obtained, as the value of air entry value at higher net stress levels can be directly obtained from soil water retention curves experimentally measured by Lee et al. (2005) at higher net stress levels. For this purpose, the Brooks–Corey model (Eq. 48) has been used to simulate the experimental data and to obtain the air entry value. The resulting effective stress parameter for weathered granite obtained from the proposed equation (Eq. 67) is depicted in Fig. 2. Table 1 presents the values of different parameters required for predicting the effective parameter for weathered granite. The air entry values in this approach were all determined directly from the available experimental data, and the slope of SWRC was considered to be almost the same under different net stress levels (consistent with the assumptions in the previous studies [see, e.g. Tarantino (2009)]. Therefore, the coupling parameter was the only parameter which was found from calibrating the proposed equation with the experimental data at zero net stress level. Then for higher net stress levels, the proposed equation was evaluated, which resulted in a fairly well agreement with the experimental data. For Kidston tailing and Talybont clay, as the parameters of soil water retention curve at higher net stress levels were not available in the experimental results, a systematic approach was employed. For Kidston tailing, first the slope of soil water retention curve was determineddirectly from soil water retention curve measured at zero net stress level. Then, the slope of the SWRC and the proposed equation for the effective stress parameter were used to determine the air entry value at 30 kPa and coupling term. Having the coupling term, and the slope of soil water retention curve, then for higher net stress levels, namely, 125 and 250 kPa, the sole parameter which needed to be found was air entry value which could directly be obtained by the use of the proposed formula for the effective stress parameter, while the performance of the proposed equation could also be assessed (see Fig. 3; Table 2, for a comparison of the proposed equation and the experimental data for Kidston tailing as well as the values of different parameters). For Talybont clay, the values of slope of SWRC at zero net stress and the effective stress parameter at zero net stress level were used to find the coupling parameter, and then, the air entry value at 204 kPa (30 psi) was obtained by fitting the proposed equation to the effective stress values at higher net stress level. The comparison between the proposed equation and the experimental data of Talybont clay (Fig. 4, Table 3) shows some discrepancy which can root in the existence of micro- and macro-pores (a double porous structure). In order to be able to address the presence of double porous structure, the use of more advanced retention equations may be necessary which account for the presence of steps (plateaus) in the water retention curves, and accordingly in the effective stress parameter versus suction. However, the overall agreement of the proposed equation with the experimental data is good enough to consider it as a proper approach to address the variation of the effective stress parameter with the net stress and capillary pressure. It is noteworthy that the equation proposed by Khalili and Khabbaz (1998) works perfectly for Kidston tailing at different net stress levels (Fig. 3), but it shows less favourable results for weathered granite and Talybont clay. As we just discussed for Talybont clay, a double porous structure can be the possible reason for a poor prediction by both approaches. The impreciseness in prediction for weathered granite as compared to our approach roots in the empirical nature of the equation proposed by Khalili and Khabbaz (1998) and the assumptions involved.

**Table 1:** Values of different hydromechanical parameters appearing in the proposed equation (for weathered granite)

Net stress level (kPa)	$P_{\mathrm{c},\mathrm{ae}}$ (kPa)	$\lambda$ (–)	$\xi$ (–)
0	1.73	0.2899	1.5816
100	4.068	0.2899	1.5816
200	6.05	0.2899	1.5816
300	8.739	0.2899	1.5816

**Table 2:** Values of different hydromechanical parameters appearing in the proposed equation (for Kidston tailing)

Net stress level (kPa)	$P_{\mathrm{c},\mathrm{ae}}$ (kPa)	$\lambda$ (–)	$\xi$ (–)
30	16.63	0.4398	0.9014
125	32.68	0.4398	0.9014
250	52.88	0.4398	0.9014

## Conclusion

As indicated in the current study, a direct link between soil water retention curve and the effective stress formulation for unsaturated soils can be established via principles of thermodynamics and mixture theory. The resulting formula contains coefficients that have physical meaning and can be obtained from soil water retention curve. Because hydromechanical coupling is introduced through a theoretical framework, the stress-level dependency of soil water retention curve is explicitly taken into account in the resulting effective stress formulation and one does not need to heuristically examine its coefficients for new conditions. It is, therefore, superior to current formulations in the literature, which are empirically obtained. The proposed equation holds as long as its assumptions hold, and thus, its scope of application can be easily explored, whereas for the empirical equations of the effective stress parameter, it is difficult to notice the range of their coefficients, physical meaning and connection to other parameters and variables. It is noteworthy that the proposed thermodynamic framework allows us to connect the effective stress equation to that of soil water retention curve in each hydraulic path (i.e. wetting, drying and scanning) which means theoretically it can capture the hydraulic hysteresis. In this study, we, however, focused on the variation of effective stress parameter in one hydraulic path. Future study is recommended to examine the efficacy and applicability of the proposed equation in different hydraulic paths. Last but not the least, the presence of disconnected phases and/or interfaces needs to be taken into account in most general case which we neglected in this study to simplify the framework. Accounting for hydromechanical coupling while considering the presence of all interfaces and phases will be a next step complementing this study. Of course, in order to be able to look into different phases and interfaces and their variation under different loading conditions, further progress in the current experimental techniques is essential.

**Table 3:** Values of different hydromechanical parameters appearing in the proposed equation (for Talybont clay)

Net stress level (kPa)	$P_{\mathrm{c},\mathrm{ae}}$ (kPa)	$\lambda$ (–)	$\xi$ (–)
0	7	0.1887	3.1846
204	20.78	0.1887	3.1846

## List of symbols

$\varvec{D}^\alpha$: Deformation rate tensor of constituent $\alpha$
$\frac{D^\alpha }{Dt}$: Time derivative for an observer fixed to constituent $\alpha$
$\varvec{E}$: Green strain tensor of the solid
$F^\alpha$: Free energy of constituent $\alpha$ per unit mass of constituent
$\varvec{F}$: Deformation gradient tensor of the solid
$$\varvec{I}$$: Unity tensor
*J*: Determinant of the deformation gradient tensor $\varvec{F}$
*p*: Pressure
$$p_{\mathrm{air}}$$: Air pressure
$$p_\mathrm{c}$$: Capillary pressure
$p_{\mathrm{c},\mathrm{ae}}$: Air entry pressure
$R^\alpha$: Lagrangian apparent density of constituent $\alpha$
*S*: Saturation
$\varvec{v}^\alpha$: Velocity of constituent $\alpha$
*W*: Free energy of the mixture per unit initial volume
$\mu ^\alpha$: Chemical potential constituent $\alpha$
$\mathbf {\pi }^\alpha$: Momentum interaction of constituent $\alpha$
$\phi ^\alpha$: Eulerian volume fraction of constituent $\alpha$
$\varPhi ^\alpha$: Lagrangian volume fraction of constituent $\alpha$
$\psi ^\alpha$: Free energy of constituent $\alpha$ per unit current volume of mixture
$\chi$: Effective stress parameter
$\rho ^\alpha$: Apparent density of constituent $\alpha$
$\rho _\mathrm{i}^\alpha$: Intrinsic density of constituent $\alpha$
$$\varvec{\sigma }$$: Stress tensor
$\varvec{\sigma }^\mathrm{s}$: Partial stress tensor of solid
$\varvec{\sigma }^\mathrm{w}$: Partial stress tensor of wetting fluid
$\varvec{\sigma }^{\mathrm{nw}}$: Partial stress tensor of non-wetting fluid
$\varvec{\sigma }_{\mathrm{eff}}$: Effective stress tensor of the mixture
$\varTheta$: Effective degree of saturation
